# Targeted prevention in primary care aimed at lifestyle-related diseases: a study protocol for a non-randomised pilot study

**DOI:** 10.1186/s12875-018-0820-8

**Published:** 2018-07-21

**Authors:** Lars Bruun Larsen, Anders Larrabee Sonderlund, Jens Sondergaard, Janus Laust Thomsen, Anders Halling, Niels Christian Hvidt, Elisabeth Assing Hvidt, Troels Mønsted, Line Bjornskov Pedersen, Ewa M. Roos, Pia Vivian Pedersen, Trine Thilsing

**Affiliations:** 1 Department of Public Health, Research Unit of General Practice, University of Southern Denmark, Odense, Denmark; 2 Department of Clinical Sciences, Center for Primary Health Care Research, Lund University, Lund, Sweden; 3 Department of Informatics, University of Oslo, Oslo, Norway; 4 Department of Business and Economics, COHERE, University of Southern Denmark, Odense, Denmark; 5 Department of Sports Science and Clinical Biomechanics, Research Unit for Musculoskeletal Function and Physiotherapy, University of Southern Denmark, Odense, Denmark; 6 National Institute of Public Health, University of Southern Denmark, Copenhagen, Denmark

**Keywords:** Targeted health checks, Primary care, Intersectoral collaboration

## Abstract

**Background:**

The consequences of lifestyle-related disease represent a major burden for the individual as well as for society at large. Individual preventive health checks to the general population have been suggested as a mean to reduce the burden of lifestyle-related diseases, though with mixed evidence on effectiveness. Several systematic reviews, on the other hand, suggest that health checks targeting people at high risk of chronic lifestyle-related diseases may be more effective. The evidence is however very limited. To effectively target people at high risk of lifestyle-related disease, there is a substantial need to advance and implement evidence-based health strategies and interventions that facilitate the identification and management of people at high risk. This paper reports on a non-randomized pilot study carried out to test the acceptability, feasibility and short-term effects of a healthcare intervention in primary care designed to systematically identify persons at risk of developing lifestyle-related disease or who engage in health-risk behavior, and provide targeted and coherent preventive services to these individuals.

**Methods:**

The intervention took place over a three-month period from September 2016 to December 2016. Taking a two-pronged approach, the design included both a joint and a targeted intervention. The former was directed at the entire population, while the latter specifically focused on patients at high risk of a lifestyle-related disease and/or who engage in health-risk behavior. The intervention was facilitated by a digital support system. The evaluation of the pilot will comprise both quantitative and qualitative research methods. All outcome measures are based on validated instruments and aim to provide results pertaining to intervention acceptability, feasibility, and short-term effects.

**Discussion:**

This pilot study will provide a solid empirical base from which to plan and implement a full-scale randomized study with the central aim of determining the efficacy of a preventive health intervention.

**Trial registration:**

Registered at Clinical Trial Gov (Unique Protocol ID: TOFpilot2016). Registered 29 April 2016. The study adheres to the SPIRIT guidelines.

**Electronic supplementary material:**

The online version of this article (10.1186/s12875-018-0820-8) contains supplementary material, which is available to authorized users.

## Background

In this paper we report on a non-randomized pilot study examining the efficacy of a preventive healthcare intervention. The intervention has been designed to systematically identify patients at high risk of developing lifestyle-related disease, and provide targeted and coherent preventive services to these individuals [1].

Lifestyle-related disease refers to health conditions that are predominantly caused by health-risk behaviors, such as poor diet, smoking, high consumption of alcohol, or lack of exercise. The consequences of lifestyle-related disease represent a major challenge for the individual as well as for society at large [2]. In Denmark, people who smoke tobacco, consume excessive amounts of alcohol, and have a sedentary lifestyle are nearly seven times as likely to die from lifestyle-related diseases than physically active non-smokers with a moderate intake of alcohol [3]. It is estimated that 80% of cardio-vascular disease (CVD), type 2-diabetes mellitus (T2DM), and chronic obstructive pulmonary disease (COPD), and 40% of all cancers may be averted by maintaining healthy dietary habits, regularly exercising, and refraining from smoking [4]. Indeed, preventable lifestyle-related diseases account for approximately 50 to 60% of all hospital admissions [5]. It is expected that increasing rates of obesity and physical inactivity will lead to a surge in the number of patients with lifestyle-related diseases in the decades to come [6–8]. In light of these trends, there is a substantial need to advance and implement evidence-based health strategies and interventions that facilitate the identification and management of people at risk of developing these diseases [9].

Disease prevention is a central task in general practice in Denmark and the Nordic countries [10]. Two recent systematic reviews of general practice health checks suggest that people at high risk of chronic disease may benefit from targeted preventive health checks [11, 12]. Indeed, targeted, or selective, preventive healthcare is a generally accepted and well-integrated part of healthcare systems worldwide (e.g. treatment of hypertension and hyperlipidemia). Other studies, however, suggest that systematic screening of the general population does not improve clinical endpoints above and beyond those associated with opportunistic screening. These studies indicate that, at a population level, systematic screening of the general population does more harm than good [13–15]. Overall, however, the evidence on targeted and systematic screening of chronic disease is very limited, possibly providing an explanation for the apparent contradictions in the literature. To this end, projects in the Netherlands and Great Britain are currently underway, testing different approaches to targeted and systematic intervention in general practice [16, 17].

There is an even greater lack of evidence when it comes to targeted preventive interventions that comprise both general practice and community health services. In such an approach the general practitioner (GP) targets patients at high risk for lifestyle-related diseases and engages in risk-management of biomarkers and disease with behavior change and pharmaceutical interventions when needed. Community health services, on the other hand, focus primarily on the prevention of health-risk behaviors - including tobacco use, poor diet, excessive alcohol consumption, and sedentary lifestyles - and provide behavior-change interventions such as smoking cessation assistance and dietary advice. Danish studies suggest a potential to enhance the collaboration and cohesiveness of the various components that comprise the preventive healthcare services in the Danish primary care system – especially between GPs and community health services [18, 19]. Outside of the Danish context, the benefits of a more unified and coherent healthcare service have also been advanced in peer-reviewed studies [20, 21]. However, effectiveness studies of a unified approach, such as that described above, seem to be lacking.

In 2012, we carried out a feasibility study, testing a novel approach to population-based risk stratification at four Danish GP clinics [22]. The intervention combined lifestyle survey data with health record information in order to identify presumably healthy individuals who nonetheless were at high risk of developing lifestyle-related diseases. These individuals were then offered a health check at their GP for a more definitive assessment of their general health as well as their risk of developing lifestyle-related diseases. Results indicated that this approach to preventive action was indeed feasible, and thus ultimately inspired the development of a large randomized study, the present TOF-project (TOF is a Danish acronym for Early Detection and Prevention). The principal aim of the upcoming TOF-project is to examine the efficacy of a preventive healthcare intervention that systematically identifies individuals at high risk of lifestyle-related disease, and provides targeted and coherent preventive services. We expect that significant changes in the targeting and systematization of disease prevention in the Danish primary care sector, including earlier detection and more coherent preventive services, will diminish the individual and societal burden of chronic disease. Due to the complexity of the TOF intervention, and the relatively high number of stakeholders, a pilot study needs to be conducted before full-scale implementation and evaluation [23]. The aim of the pilot study is to test the acceptability, feasibility, and short-term effects of a selective preventive program, designed to systematically help patients evaluate their individual risk of lifestyle-related disease. The program also offers targeted and coordinated preventive services in the primary healthcare sector.

## Methods

The pilot study was designed as a population based non-randomized study in the Region of Southern Denmark, comprising 22 municipalities, 787 GPs, and a general population of 1,2 million. The Danish health care system is a tax-based system comprising three levels: A national level responsible for, among other things, public health, planning, and patient safety; a regional level responsible for the hospitals and the primary care sector; and a municipal level responsible for primary prevention, rehabilitation, and patient education. General practice and the municipalities have shared responsibility for preventive services aimed at the individual. Specifically, GPs assess patient health and implement disease-specific secondary prevention. The municipalities, however, are tasked with primary prevention such as smoking cessation, alcohol treatment, and other lifestyle related services. GPs are organized in clinics with an average of two GPs per clinic. While most clinics comprise a single GP, some have up to ten. Almost all Danish citizens (98%) are registered with a GP [24, 25]. Each GP has an average of 1600 registered patients.

### Recruitment

The pilot study targets adults born between year 1957 and 1986. All 22 municipalities in the Region of Southern Denmark were invited to participate in TOF. Ten municipalities (Esbjerg, Haderslev, Varde, Sønderborg, Aabenraa, Middelfart, Kerteminde, Nyborg, Svendborg, Langeland) submitted expressions of interest to participate in the study, and were approved for participation by the Regional Council. Two of the municipalities (Haderslev and Varde) volunteered to participate in the pilot study.

The municipalities of Haderslev and Varde comprise 55,971 and 50,110 citizens, and 37 and 29 GPs, respectively. All GPs from each municipality were invited to an information meeting before being formally invited to participate in the pilot study. The invitation was followed up with telephone calls to the individual GP clinics. All patients were invited at baseline, and the intervention was taken up by the patients at their own convenience during the intervention period. See Additional file 1 for a more detailed project flow showing the recruitment, intervention and evaluation phases.

### Organization and development of the intervention

The intervention was planned during a two-year combined effort involving all stakeholders. End users were involved in the design of the intervention, including patients, GPs, and municipal health professionals. A group of seven GPs developed the targeted intervention at a general practice level during five workshops. Similarly, a group of 10 municipal health workers, one from each of the participating municipalities, developed the targeted intervention at a municipal level during 10 workshops. The workshops lasted between 2 h and 2 days. A digital support system was created and tested by user populations, including patients, non-government patient organizations, GPs, and municipal health professionals.

A steering committee was established at the start of the project, consisting of managers or board members from the Region of Southern Denmark (project owner), The Organization of General Practitioners in Denmark (PLO), the 10 participating municipalities, the Research Unit for General Practice at the University of Southern Denmark (FEA), and the Danish Quality Unit for General Practice (DAK-E). The chair of the committee is the health director from the Region of Southern Denmark. A research committee with participation from the steering committee chair and the primary investigator has been established. A mission statement has been approved by the steering committee and an agreement of co-operation has been signed between the Region of Southern Denmark and the University of Southern Denmark. The agreement states that the University of Southern Denmark holds all rights, intellectual as well as judicial, to the research data, and that the Region of Southern Denmark has no right to oppose publication of results. The research committee approves all access to research data from affiliated researchers.

Prior to study commencement, all enrolled GPs, practice nurses (PN), and health professionals from the municipalities were invited to a joint three-hour training course (August 2016). The course focused on the assigned intervention activities and tasks both within the GP clinics and the municipality respectively, and between GPs and the municipality.

### Invitation and consent

The source population received an invitation to participate, sent on behalf of the GP and the municipality to the individual’s digital mailbox. All permanent residents in Denmark are obligated to have a digital mailbox, which is essentially a digital mail-system provided by the government for secure and direct communication between individuals and public authorities and other trusted organizations (e.g. banks and insurance companies) [26]. People may opt out of the digital mail system, citing low IT-literacy (usually elderly persons), cognitive impairment, or other complicating factors. To enroll in the study, individuals were asked to follow a link in the invitation to a digital support system protected by a two-phased NemID password [27]. NemID is a password system providing an exact identification of the user. This system is utilized by Danish public and non-public institutions to provide secure access to personal information, such as health and financial data. Through digital mail and NemID, we were able to reach and identify 97% of the target population. In April 2016, participants received an invitation with an embedded hyperlink to a digital consent form on a secure webpage in their digital mailbox. The consent form outlined study participation and disclosure of data from the GPs electronic patient record (EPR) and was supplemented with short videos describing the purpose of the study and the intervention. Participants were asked to read the information and electronically sign the consent form. Two reminders were sent after one and 2 weeks if participants failed to sign the form. Enrollment closed after 6 weeks. At this time, information on relevant diagnosis (International Classification of Primary Care (ICPC-2) codes) and prescribed medicine (Anatomical Therapeutic Chemical Classification (ATC) codes incl. Text fields with indication for treatment) were collected from the GPs EPR system (See Table 1 for the ICPC-2 codes and ATC codes that were accessed based on the consent). Five months after consent (September 2016), participants received another digital invitation in the digital mailbox, this time to fill in a questionnaire and access a personal health profile. Participants could opt-out at any time during the intervention period by clicking an “opt-out” button on the digital support system.

**Table 1:** The 5As model

Assess	Initial questionnaire-derived assessment of the patients’ health / risk profile for the purpose of identifying patients in need of health-risk behavior change Subsequent health examination at the GP to confirm or disprove estimated risk of disease
Advice	Counseling, based on the patient’s symptoms / risk profile. The patient’s values and attitudes can usefully be involved
Agree	Active involvement of the patient in connection with goal setting regarding health-risk behavior change
Assist	Joint development of plan for health-risk behavior change
Arrange follow-up	Planning of the next steps at the GP or other (e.g. the municipality)

### Intervention

The duration of the intervention was 3 months and took place between September 2016 and December 2016. The intervention comprised a two-pronged approach: [1] a joint intervention applied to the entire sample, regardless of whether the participants were healthy, at risk, or already in treatment for T2DM, COPD, CVD, hypercholesterolemia or hypertension [2] a targeted intervention that was offered only to participants who presumably would benefit from either further examinations at the GP (high risk), or from receiving community health services, such as smoking cessation, dietary advice, or physical activity (health-risk behavior).

*The joint intervention consisted of:*

- Stratification to one of four risk groups. Stratification to a specific risk group was determined by use of risk algorithms and EPR information
- A digital support system with user interfaces for all users, including the patient, the GP, and the municipal health professional
- An individual health profile

*The targeted intervention consisted of:*

- A focused clinical examination and a subsequent health dialogue with a GP (targeting patients at high risk), *and / or*
- A short telephone-based health dialogue with a municipal health professional. For patients with limited capability to care for their own health, this initial talk could be followed up with a subsequent face-to-face health dialogue (targeting patients with health-risk behavior)

For all present intents and purposes, the term *health dialogue* refers to a consultation that includes the elements of the 5As model (see Table 1) and the techniques used in motivational interviewing [28, 29].

### The joint intervention

All participants gained access to the digital support system and were invited to fill in a questionnaire. The participant questionnaire contained 15 items on height, weight, self-perceived health status, family history of lifestyle-related diseases, COPD related symptoms, smoking status, leisure activity level, alcohol consumption, diet, and osteoarthritis risk factors. Questions about family history of diabetes and leisure activity level were taken from the Danish Diabetes Risk model [30]. Similarly, questions on COPD-related symptoms and smoking status were derived from the COPD-PS screener [31] and the Heartscore BMI score [32]. Items tapping dietary habits were from the Swedish National Guidelines on Disease Prevention [33]. The questionnaire took approximately 5 min to complete.

Based on the questionnaire and information from the individual EPR, participants were stratified into four distinct risk groups:

- *Group 1* – Participants with a pre-existing diagnosis and/or in current treatment for a lifestyle-related disease.
- *Group 2* – Participants at high risk of developing lifestyle-related disease, and thus eligible for the offer of a targeted intervention at the GP.
- *Group 3* – Participants engaging in health-risk behavior, and thus eligible for the offer of a targeted intervention at the municipality.
- *Group 4* – Participants with a healthy lifestyle and no need for further intervention.

### Stratification to group 1

EPR data was collected via certified EPR-suppliers. We used International Classification of Primary Care-2 codes (ICPC-2) registered by the GP and/or Anatomical Therapeutic Chemical Classification **(**ATC) codes for prescribed medicine within the past 2 years, together with the indication for prescribing the medicine, to identify Group 1 participants (see Table 2).

**Table 2:** Criteria for identification of participants with a pre-existing diagnosis and/or in current treatment for a lifestyle-related disease

Diagnosis	Diagnostic code(s) (ICPC-2)		ATC therapeutic code(s) for prescribed medicine and indicative texts for the prescription^a^
Hypertension	K86, K87	or	C0 *BT*, *bt*, *Bt*, *ypert*, *ldot*, *LODTR*, *lotr*, *lodptr*, *blodtryk* *bl. trykket*, *lodrtr*^b^
Hyperlipidemia	T93	or	C10 *kolesterol*
COPD	R95	or	R03AC18 (indacaterol), R03AC19 (Olodaterol), R03AL03 (vilanterol), R03AL04 (Indacaterol+Glycopyrroniumbromide), R03AL05 (Formoterol+Aclidiniumbromide) R03BB04 (tiotropium bromide), R03BB05 (Aclidiniumbromide), R03BB06 (Glycopyrroniumbromide)*obstruktiv*, *KOL*
T2DM	T90	or	A10(diabetes medicine) *sukkersyge*, *diabetes*
CVD	K74, K76^c^		

^a^The indicative text is in Danish and has not been translated as some indicative texts are only parts of the entire word and as such not translatable

^b^The reason for the large number of indicative text is misspellings by the GP when indicating the purpose of the prescription

^c^Diagnostic codes for ischemic heart diseases are transferred to the GP’s EPR system when the patients are discharged from the hospital following an angina or a stroke. ATC codes for prescribed medicine will not provide further information

Given the pre-existing diagnosis and/or treatment, Group 1 was excluded from the subsequent risk estimation and stratification into Group 2, 3, and 4.

### Stratification to group 2

Next, participants at risk of lifestyle-related disease were identified using three validated risk scores: the Chronic Obstructive Pulmonary Disease Population Screener (COPD-PS), the Danish Diabetes Risk model, and a modified Heartscore BMI score [30–32]. The COPD-PS uses an algorithm accounting for age, lifetime use of cigarettes, and smoking-related symptoms to identify at-risk patients who may benefit from a spirometry to test for COPD (Table 3) [31]. The Danish Diabetes Risk score is based on an algorithm that incorporates age, sex, BMI, known hypertension, leisure activity level, and family history of diabetes (Table 4) [30]. The modified Heartscore BMI score accounts for age, sex, body mass index (BMI), and smoking status (Table 5) [32].

**Table 3:** Algorithm used for risk assessment of COPD

Characteristic		Score
During the past 4 weeks, how much of the time did you feel short of breath during every day activities? (e.g. Strolling, light gardening, cleaning, shopping etc.)	None of the time	0
	A little of the time	0
	Some of the time	1
	Most of the time	2
	All of the time	2
Do you ever cough up any “stuff,” such as mucus or phlegm?	No, never	0
	Only when I have a cold, pneumonia or sore throat	0
	Yes a few days a month	1
	Yes most days a week	1
	Yes every day	2
Please select the answer that best describes you in the past 12 months. I do less than I used to because of my breathing problems.	Strongly disagree	0
	Disagree	0
	Unsure	0
	Agree	1
	Strongly agree	2
Have you smoked at least 100 cigarettes in your ENTIRE LIFE?	Yes	2
	No	0
Age	35–49 years	0
	50–59 years	1
	60–69 years	2
	+ 70 years	2

Cut off value: ≥5

**Table 4:** Algorithm used for risk assessment of T2DM

Characteristic	Score
Sex	
Male	1
Female	0
Age	
40–44 years	0
45–49 years	1
50–54 years	2
55–59 years	3
60–69 years	4
BMI	
25–30 kg/m^2^	1
> 30 kg/m^2^	2
Known hypertension	
Yes	2
No	0
Primary recreational activity level during the past year:	
Participating in sports competitions or hard exercise several times a week	0
Active with sports at least three times a week or regularly perform heavy house or garden work	0
Strolling, cycling or other light exercise at least 4 h a week (including Sunday walks, light gardening and cycling/walking to work)	1
Reading, watching television or other sedentary jobs	1
Family history of diabetes (Family includes grandparents, parents, sibling and children):	
No family member with diabetes before the age of 70	0
One family member with diabetes before the age of 70	1
More than one family member with diabetes before the age of 70	2
Having had diabetes including gestational diabetes	
Yes	2
No	0

Cut off value: ≥5

**Table 5:** Algorithms used for risk assessment of CVD

Age	BMI	Daily smoker
Female		
> 50 years	> 40 kg/m^2^	+
> 55 years	> 40 kg/m^2^	–
> 58 years	35–40 kg/m^2^	+
Male		
> 49 years	> 40 kg/m^2^	+
> 55 years	> 40 kg/m^2^	–
> 50 years	35–40 kg/m^2^	+
> 55 years	35–40 kg/m^2^	–
> 52 years	30–35 kg/m^2^	+
> 56 years	25–30 kg/m^2^	+

Consistent with the criteria of the four distinct stratification groups defined above, participants were categorized into Group 2 when one or more of the risk assessment algorithms indicated high likelihood of developing lifestyle-related disease (see Tables 3, 4 and 5).

### Stratification to group 3 and 4

Finally, participants engaging in health-risk behavior with one or more risk factors were categorized in Group 3 (Group 3). Health-risk behavior was defined by the presence of at least one of the following behaviors: smoking tobacco on a daily basis, consuming more than 14/21 (male/female) standard units of alcohol per week, sustaining an unhealthy diet (diet score ≤ 4 on a 12-point score drawn from the Swedish National Guidelines on Disease Prevention) [4], maintaining a BMI ≥ 35, and/or engaging in a generally sedentary lifestyle. Lastly, participants with no lifestyle-related disease or risk thereof were stratified into Group 4.

### Digital support system

All users had access to a digital support system in the form of a web page with a common database and specific user interfaces for the GP, the municipality health professionals, and the patient. No apps were developed. The system design drew inspiration from the work by Krist and colleagues’ research on preventive EPRs, and was further inspired by the results of a Delphi process carried out to identify factors for optimal development of health-related websites [34–36]. Due to challenges in terms of interoperability between the eight suppliers of EPR systems used by GPs, and at least three suppliers of electronic care records (ECR) in the municipalities, it was not feasible to develop a support system that completely integrated the EPR and ECR systems. Instead, the digital support system was developed as a parallel system with an additional functionality facilitating the transfer of information (e.g. relating to lifestyle and/or prevention plans) to the EPR and ECR systems using Electronic Data Interchange (EDIfact) messages [22]. The patient controlled access to personal health information on the system, such that the GP and municipal health professional were only able to access this information with the explicit consent of the patient.

**Fig. 1:**
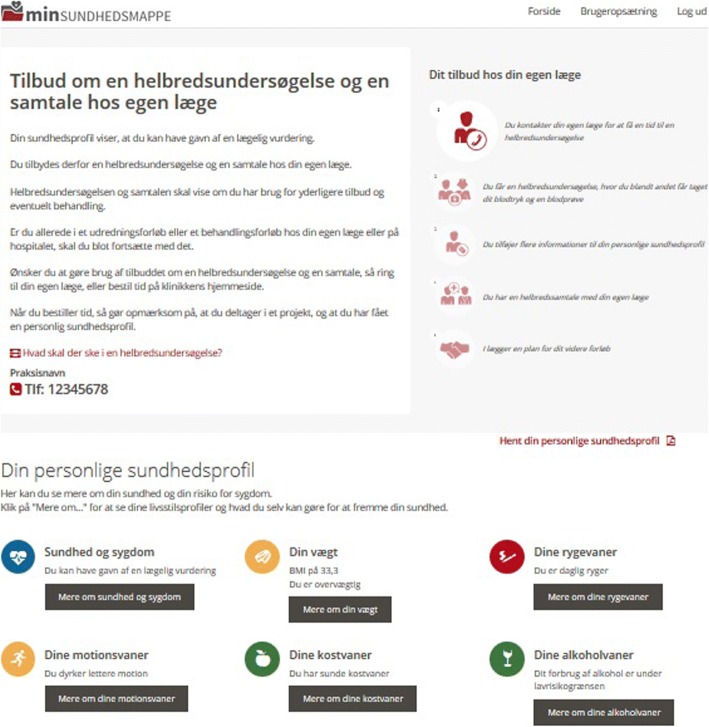
Screen dump from digital support system (in Danish)

The digital support system was developed iteratively in collaboration with the users during the before mentioned workshops with municipality health professionals and GPs and in the form of usability tests with patients. The user interface for the patient was responsive and compatible with most devices, including mobile phones, tablets, laptops and stationary computers. Due to technical constraints in the secure log-in provided by NemID, the user interface for health professionals was only developed for laptops and stationary computers. In order to make the user interface for the patient as intuitive and user-friendly as possible, the digital support system made extensive use of simple visualizations, icons, and short information videos (Fig. 1). The primary text-based messages were kept short and concise with the provided possibility of accessing secondary in-depth information, retrieved from the Danish Health Portal, sundhed.dk [37].

Beyond facilitating the intervention, the digital support system also enabled data collection for research purposes. A number of questionnaires were sent from the digital support system to the participants at specific time-points, including immediately after consent, after receiving the personal health profile, following the health dialogue at the GP, and at the end of the implementation period. Questionnaire reminders were sent by e-mail with a link to the digital support system. The GPs and municipal health professionals received audits in the form of short questionnaires immediately after each consultation as well as before and after the study period (GPs only).

### Personal health profile

Based on results of the stratification process, each patient received a personal health profile on the digital support system. The purpose of the health profile was to encourage patients to change their health-risk behavior and follow the tailored advice provided by the system. Patients who were at increased risk of developing a lifestyle-related disease (Group 2) were advised to consult their GP for further examination and advice. Similarly, patients engaging in health-risk behavior (Group 3) were offered lifestyle counseling, or lifestyle courses from the municipality health services. By definition, Group 4 patients lead a relatively healthy life with no need for health-risk behavior change. Group 1 patients were advised to continue their treatment and use the information provided to change health-risk behavior.

The personal health profile included individualized information on current health-risk behavior and risk of disease. The information was tailored based on the questionnaire, the information from the EPR, and the risk scores on COPD, T2DM, and CVD. It also included general health information and information about preventive health services concerning smoking, diet, exercise, and alcohol consumption. This information was provided by the municipality, the Region of Southern Denmark, or national health services, and targeted the individual (e.g. via links to apps and webpages) based on his/her specific health-risk behavior.

### The targeted intervention

#### The intervention at the GP

The intervention at the general practice level consisted of a focused clinical examination and a subsequent health dialogue and was offered to patients who were at increased risk of developing a lifestyle-related disease (Group 2). Group 2 patients accepted the offer of the intervention by scheduling an appointment at the GP (either by phone or the GP’s webpage). Whether the patient participated in the intervention or not was thus determined by their motivation and capabilities as well as the extent to which the content of the personal health profile motivated the patient to take action. The intervention was applied within the framework of the 5As model (see Table 1) [28]. The content of the focused clinical examination was based on the patient’s health profile, and might include measurements of blood glucose (HbA1c) and cholesterol levels, as well as height, weight, blood pressure, and lung function measurements and Electrocardiogram (ECG). Results from the examinations were registered in the digital support system where both the patient and the GP could access them at any time. After the focused clinical examination all patients were given the opportunity to prepare for the subsequent health dialogue by answering a questionnaire inspired by three systematic reviews on the determinants of behavior change [38–40]. These included questions about motivation, resources, former experiences with behavior change, social network, mental health (WHO-5 for stress and Major Depression Inventory (MDI) for depression) [41, 42], and a scheme to qualitatively self-report on facilitators and barriers to behavior change (a so-called balance-sheet). The questionnaire results were shared with the GP on the digital support system. Based on the health dialogue, the GP and the patient developed a prevention plan that included a goal, a time frame, and identification of the appropriate means to fulfill the plan (e.g. reference to a smoking cessation course, or follow-up at the GP). The prevention plan was registered on the digital support system by the GP and was accessible to both the GP and the patient.

#### The intervention at the municipal level

The intervention at the municipal level was offered to patients exhibiting health-risk behavior (Group 3) and consisted of a short telephone consultation with a health professional – for example a nurse, a dietician, or a physiotherapist. A subsequent face-to-face health dialogue was offered to patients who were deemed to potentially benefit from more extensive support. Group 3 patients requested the intervention on the digital support system by filling in a short form and sending it by e-mail to the municipality. A municipal health professional would then call the patient within the following week. Similar to the GP intervention, the intervention at the municipal level was thus also determined by patient motivation and capabilities as well as the extent to which the content of the personal health profile motivated the patient to take action. Immediately after the intervention, a participation form was sent to the municipality. Patients could prepare for the upcoming call from a municipal health professional in the same way as Group 2 patients prepared for the health dialogue – that is, by answering a short questionnaire. Ultimately, a prevention plan, including concrete details on its execution, was developed based on the telephone consultation and the face-to-face health dialogue. The prevention plan was registered by the municipal health professional and presented on the user interfaces of both the municipality and the patient.

### Sample size calculation

While aiming to test the acceptability, feasibility, and short-term effects of the pilot, we estimated a sample size for each GP that would allow the GP to familiarize him/herself with the intervention without unnecessary increases in workload during the intervention period. In agreement with the GP representative in the Region of Southern Denmark, we set a target of four health checks for each GP. From the feasibility study, we estimated that 60% would consent to the study, and that 75% of these participants would receive a personal digital health profile [22]. Also based on the feasibility study, we estimated that 12% of the study population would be recommended to consult their GP (Group 2). From results obtained in similar Dutch studies, we finally estimated that 35% of the these patients (Group 2) would eventually consult the GP [43]. Given these figures, we calculated that a total sample of approximately 200 patients from each GP would be required to reach the target of four completed health checks per GP.

### Data collection and analysis

#### Evaluation outcomes

Evaluation of the study will be carried out using quantitative as well as qualitative research methods (Table 6). All outcome measures are based on validated instruments and aim to provide results pertaining to intervention acceptability, feasibility, and short-term effects. In addition, outcomes related to other associated topics will be included. The specific instruments used will be described in detail in later publications.

**Table 6:** Outcomes

Outcome	Data input
Change in proportion of patients at increased risk of lifestyle related disease from baseline to the 12 weeks follow up	Questionnaire at baseline (Q2) and end of study period (Q6 and Q7). Risk of lifestyle related disease is based on the algorithms previously described in the methods section and in Tables 3, 4 and 5
Determinants of participation and non-participation	Questionnaire at baseline (Q2). Participants and non-participants will be compared with regard to socio-demographic characteristics, morbidity and contextual characteristics
Evaluation of the digital support system with focus on design, usability and effect of the decision support system	Focus group interviews before study start comprising 6 GPs, 6 practice staff members, 6 municipality staff members, 6–8 patients and representatives from 6 to 8 stakeholder organizations, respectively
	Qualitative interviews with 8–10 patients before and after the health dialogue at the GP, and qualitative interviews with 6–8 GPs after health dialogues, focusing on the experienced usefulness of the digital support system
	Questionnaire to all participating patients immediately after signing the consent form (Q1) and after receiving the health profile (Q3 and Q4) and to all participating GPs and municipality staff members following each study related patient encounter (Q8-Q13)
Process evaluation focusing on the intervention in general practice and the municipality	Participant observation of 10–15 health dialogues in different general practices, followed by qualitative interviews with the participating patients, GPs and practice staff
	Focus group interviews with 6–8 municipality staff members involved in the study and interviews with 10–15 patients who have attended a health dialogue in the municipality
	Questionnaire to all participating GPs, practice staff members and municipality staff members following each study related patient encounter (Q8-Q13)
Process evaluation focusing on the organizational basis of the pilot implementation	Interviews with stakeholders involved in the planning, implementation and evaluation of the study; GPs, practice staff members, municipality staff members, patients, project leaders, researchers
Process evaluation focusing on the common training course for enrolled GPs, practice staff and health professionals from the municipalities	Interviews with GPs and municipal health professionals and questionnaire at the end of the course
GP and patients preferences with regard to the content of the health dialogue, and change in preferences during the study period	Questionnaire using discrete choice modelling (Q4, Q7, Q8, Q11)
Patients’ perceptions of relational empathy following the health dialogue at the GP	Questionnaire including The Consultation And Relational Empathy (CARE) measure following each behavior counseling session in general practice (Q5)
Quality of Life Subscale on the Hip injury and Osteoarthritis Outcome Score (HOOS)/Knee Injury and Osteoarthritis Outcome Score (KOOS)	Questionnaire at baseline (Q2). Participants replying “yes” to any of the osteoarthritis related questions on hip/knee pain, GP care seeking or surgery at baseline will receive additional questions on knee/hip related quality of life and mechanical alignment of the leg and foot [55]
Patient enablement following the health dialogue at the GP	Questionnaire with Patient Enablement Instrument (PEI) (Q5)
Patient reported 1. Meaning-Making and Health, 2. Spiritual Wellbeing, 3. Religious belief and practices	Questionnaire items are sampled from the validated questionnaire SoMe (Sources of Meaning) and European Value Study (EVS) (Q6 and Q7)
GP reported 1. Perceived importance of communication on existential and spiritual issues, 2. Self-efficacy and barriers in communication on existential and spiritual issues, 3. Personal belief	Questionnaire items sampled from the validated Self-efficacy questionnaire, European Value Study (EVS) and two items developed for this study evaluation (Q6 and Q7)
Patient reported self-efficacy and change in self-efficacy during the study period as a result of participation	Questionnaire incl. The General Self-Efficacy Scale (Q3, Q4, Q6 and Q7)
Patient reported mental well-being and change in mental well-being during the study period as a result of participation	Questionnaire incl. The Warwick-Edinburgh Mental Well-being Scale (WEMWBS) (Q3, Q4, Q6 and Q7)

#### Qualitative data

Qualitative data will be derived from interviews (individual and focus groups comprising GPs, practice staff members, municipality staff members, patients from group 2 and 3, stakeholders, project leaders and researchers) and participant observations (during the health dialogues at the GP). The estimated number of participants is shown in Table 6.

#### Quantitative data

Quantitative data will be derived from questionnaires as well as Danish National registers (see section below). Table 7 shows the content of the questionnaires applied while a diagram, attached as Additional file 1, shows a flow of the entire intervention and the timing of the questionnaires during the intervention.

**Table 7:** The study questionnaires: Target groups and questionnaire items

Target group	Questionnaire	Items
Patients	Q1	Attitudes towards prevention, Risk-taking attitudes, Time preferences, Mental well-being, Self-efficacy, Evaluation of invitation and consent form
	Q2	Height, Weight, Self-perceived health status, Family history of lifestyle-related diseases, Known hypertension, COPD related symptoms, Osteoarthritis related risk factors, Smoking status, Leisure activity level, Alcohol consumption, Eating habits
	Q3	Evaluation of the personal health profile
	Q4	Evaluation of the personal health profile, Preferences with regard to the content of the health dialogue
	Q5	Patient Enablement Instrument, The Care Measurement
	Q6	Height, Weight, Self-perceived health status, Family history of lifestyle-related diseases, Known hypertension, COPD related symptoms, Osteoarthritis related risk factors, Smoking status, Leisure activity level, Alcohol consumption, Eating habits, Attitudes towards prevention, Mental well-being, Self-efficacy, Study participation, Study evaluation, Meaning-Making and Health, Spiritual Wellbeing, Religious belief and practices, Risk-taking attitudes, Time preferences
	Q7	Height, Weight, Self-perceived health status, Family history of lifestyle-related diseases, Known hypertension, COPD related symptoms, Osteoarthritis related risk factors, Smoking status, Leisure activity level, Alcohol consumption, Eating habits, Attitudes towards prevention, Mental well-being, Self-efficacy, Study participation, Study evaluation, Meaning-Making and Health, Spiritual Wellbeing, Religious belief and practices, Risk-taking attitudes, Time preferences, Preferences with regard to the content of the health dialogue
GPs	Q8	Attitudes towards prevention, Experiences with prevention, Preferences with regard to the content of the health dialogue, GPs health-risk behavior
	Q9	Content of the clinical examination, Staff and time consumption
	Q10	Evaluation of the quality of the stratification, Use and evaluation of the digital support system, The patients motivation and resources, The plan for the patient, Time consumption
	Q11	Attitudes towards prevention, Experiences with prevention, Preferences with regard to the content of the health dialogue
Municipality health professionals	Q12	Evaluation of the quality of the stratification, Use and evaluation of the digital support system, The patients motivation and resources, The plan for the patients, Time consumption
	Q13	Evaluation of the quality of the stratification, Use and evaluation of the digital support system, The patients motivation and resources, The plan for the patient, Time consumption

#### Register based data

Data from the Danish national registers concerning demographic information, prescriptions, and health care usage of the target population (*n* = 9.400) will be obtained from Statistics Denmark (https://www.dst.dk/da) [44]. Information from the different registers will be linked by the patients’ Danish Personal Identification Number.

#### Socio-demographic variables

*Information* on socio-demography encompassed educational level, occupation, income, cohabitation status, ethnicity, and residency.

*Education* is defined as the highest formal educational attainment obtained on the first of October in each calendar year.

*Occupation* is defined as the occupational status on the first of November in each calendar year.

*OECD-adjusted income level* is defined as the individual’s/family’s disposable income, adjusted for family size and categorized in relative terms (low/middle/high income) [45].

*Cohabitation* status is defined as cohabitating or living alone.

*Ethnicity* is based on country of origin and descendance.

#### Morbidity

Information on health/disease status (hypertension, hypercholesterolemia, type-2 diabetes, cardio-vascular disease) is defined in terms of ICD-10 diagnosis codes and medical usage. The ‘National Patient Registry’ will provide information on ICD-10 diagnostic codes. The ‘Register of Medicinal Product Statistics’ provide information on medical usage [46, 47].

#### Contextual variables

Contextual variables include information on study site and neighborhood social deprivation. Neighborhood social deprivation will be derived on a census district level and is principally defined in terms of the following three variables: educational attainment, employment status (employed/social welfare), and income (mean family disposable income). Educational, employment, and income deprivation thus specifically refer to the proportion of citizens within each census district who has access to basic education (up to high school), who is unemployed (e.g. students, unemployed workers), and who belongs to the lowest income quartile, respectively. Each variable is ranked, grouped in quartiles, and given a value between 0 and 3 (3 = high deprivation). This results in an aggregated ranking system ranging from 0 (low deprivation) to 9 (high deprivation). The aggregated rank is then grouped in quartiles. A neighborhood social deprivation score will be calculated for all census districts in Denmark in order to obtain local deprivation scores that mirror the relative social deprivation of the individual census district [48].

## Discussion

This pilot study will provide a solid empirical base from which to plan and implement a full-scale randomized study with the central aim of determining the efficacy of a preventive health intervention. The intervention was designed to systematically identify persons at risk of developing lifestyle-related disease or who engage in health-risk behavior, and provide targeted and coherent preventive services to these individuals.

### Strengths and limitations

Much effort has been made to define the specific nature and objective of pilot and/or feasibility studies. In a scoping review of optimization strategies for complex interventions prior to randomized trials, Levati asserts the notion that different frameworks for intervention development, such as intervention mapping and the MRC framework for complex intervention, call for different approaches to pilot and feasibility studies [49]. As a common feature when developing complex randomized trials, the authors suggest “that the acceptability of the intervention to those directly involved in the delivery and receipt of the final intervention, together with the anticipated effect of the intervention, are important elements to take into account as early as possible in the pre-trial stage.” [49].

Eldridge et al. used a Delphi survey to arrive at distinct definitions of feasibility and pilot studies [1]. They suggest that “feasibility study” is an overarching term with “pilot study” representing a subset of feasibility studies. Generally, feasibility studies ask whether something can be done, should we proceed with it, and if so, how? Pilot studies ask the same questions, but with a specific design feature of a larger study, conducted on a smaller scale. According to the authors, pilot studies can be separated in two distinct types: non-randomized and randomized. Non-randomized pilot studies do not include a control group and are usually external to the subsequent randomized controlled trial (RCT), that is, the participants are not included in the effect analysis of the RCT. Randomized pilot studies, on the other hand, randomize participants to an intervention or control group and can be internal to the subsequent RCT. Bowen et al. complement the work of Eldridge et al. and propose eight foci (design features) of feasibility studies: Acceptability, demand, implementation, practicality, adaptation, integration, expansion, and limited efficacy [50].

According to Eldridge et al., the study presented in this paper is a non-randomized pilot study. We chose a non-randomized design in order to examine the specific design features of a stepped wedge cluster randomized design for the full-scale randomized study. A stepped-wedge design is a type of cluster randomized design that meets the specific ethical and logistical demands of a delayed intervention performed in routine care where all participants will be offered the intervention [51]. The pilot resembles one cluster in a stepped wedge cluster randomized study, and will thus allow us to ascertain whether the intervention can be delivered during a three-month period, or if longer time is required to avoid carry-over effects [51]. In the event that more time is necessary to deliver the intervention, it will be difficult, if not impossible, to accurately determine the optimal duration of a cluster. This will likewise complicate the stepped wedge design. One way to compensate for incomplete knowledge on the optimal timeframe for the intervention may be to include a “wash out” period after every cluster allowing for any delay or lag in implementation before the next cluster is commenced [52]. The length of the “wash out” period can be estimated from the results of the pilot study.

We have randomly sampled 200 patients from each GP in order to have a source population that is representative of the target population. We have chosen to target people born between 1957 and 1986 to assess the risk of lifestyle-related disease and health-risk behavior at an age interval where changes in lifestyle will provide significant health effects and be cost-effective. To this end, we have chosen to assess variation in the proportion of patients at increased risk of lifestyle-related disease between baseline and the 12-week follow up as our primary health-related outcome. Further, given the fact that complex interventions, such as the one described here, usually have concurrent endpoints [23], we also collect data on a variety of other variables – both questionnaire- and register-based – related to both lifestyle and disease. We have yet to determine which of these endpoints to include in the full-scale randomized study.

We have planned the intervention in collaboration with the stakeholders, patients, and service providers in order to run a pilot study that is both acceptable and relevant for all user groups. We use quantitative as well as qualitative research methods to assess the acceptability, demand, implementation, and practicality of the pilot, from the viewpoint of both users and service-providers. In addition to evaluating the intervention, we assess the organizational challenges of planning and implementing IT-supported pilot studies [53]. At the same time we test different methods of data collection, including electronic collection of data from the digital support system and participant observations at the GP clinics. We also test various types of questionnaires, including ones that involve simple items with binary outcomes as well as others in more complex discrete choice format. The pilot will hence enable us to assess whether the intervention can be executed, and whether the organizational approach taken, fit the purpose. We will further be able to make an informed decision about how we can collect data during the full-scale study in the most efficient and cost-effective way that is also acceptable to both users and service-providers.

### From pilot to full-scale randomized study

Another issue raised by Levati et al. and Eldridge et al. concerns pinpointing the appropriate time to move from piloting to full-scale RCT. That is, should we proceed with the project, and if so, how? [1, 49]. Proceeding from pilot to a full-scale randomized study is probably the most under-researched part of the implementation of complex interventions. Bugge et al. suggest a three step process to establish the best possible foundation on which to make a decision to advance a full-scale randomized study [54]. First, any problems should be categorized into three distinct types: Issues that are likely to complicate the full-scale study, issues that are likely to complicate both trial and real-world situations, and issues that are likely to complicate real-world situations only. Next, potential solutions should be identified for the expected issues, ideally with lay participation. Finally, the best of these solutions should be selected to determine the best way to proceed. With this strategy in mind, we will do a thorough assessment of the problems encountered in the pilot before advancing the full-scale study. We will thus identify solutions in collaboration with the service providers (GPs and municipal health professionals) who participated in the pilot study, as well as with those who took part in the design of the intervention. We will also seek patient-feedback on the technical and communicative properties of the digital support system before defining its final specifications. The final assessment is presented to the steering committee that will take the decision on the way forward.

## Additional file

Additional file 1: Detailed project flow showing the recruitment, intervention and evaluation phases. Detailed project flow from recruitment to intervention and evaluation. It shows how participants will be recruited, how they will be stratified using algorithms and what intervention elements the participant will receive. Furthermore, it shows when quantitative data will be collected for evaluative purposes. (TIF 1491 kb)

## Abbreviations

ATC: Anatomical Therapeutic Chemical Classification
BMI: Body mass index
COPD: Chronic Obstructive Pulmonary Disease
COPD-PS: Chronic Obstructive Pulmonary Disease Population Screener
CVD: Cardio vascular disease
EDIfact: Electronic Data Interchange messages
EPR: Electronic patient record
GP: General practitioner
ICD-10: International classification of diseases
ICPC-2: International classification of primary care
RCT: Randomized controlled trial
T2DM: Type 2 diabetes mellitus
